# Supplementary material for: Development of a Safe and Highly Efficient Inactivated Vaccine Candidate against Lumpy Skin Disease Virus
Source: Vaccines (Basel). 2020 Dec 23;9(1):4. doi: 10.3390/vaccines9010004 (PMC7823700; doi:10.3390/vaccines9010004)
Supplement: Supplementary file 1 [file vaccines-09-00004-s001.pdf]

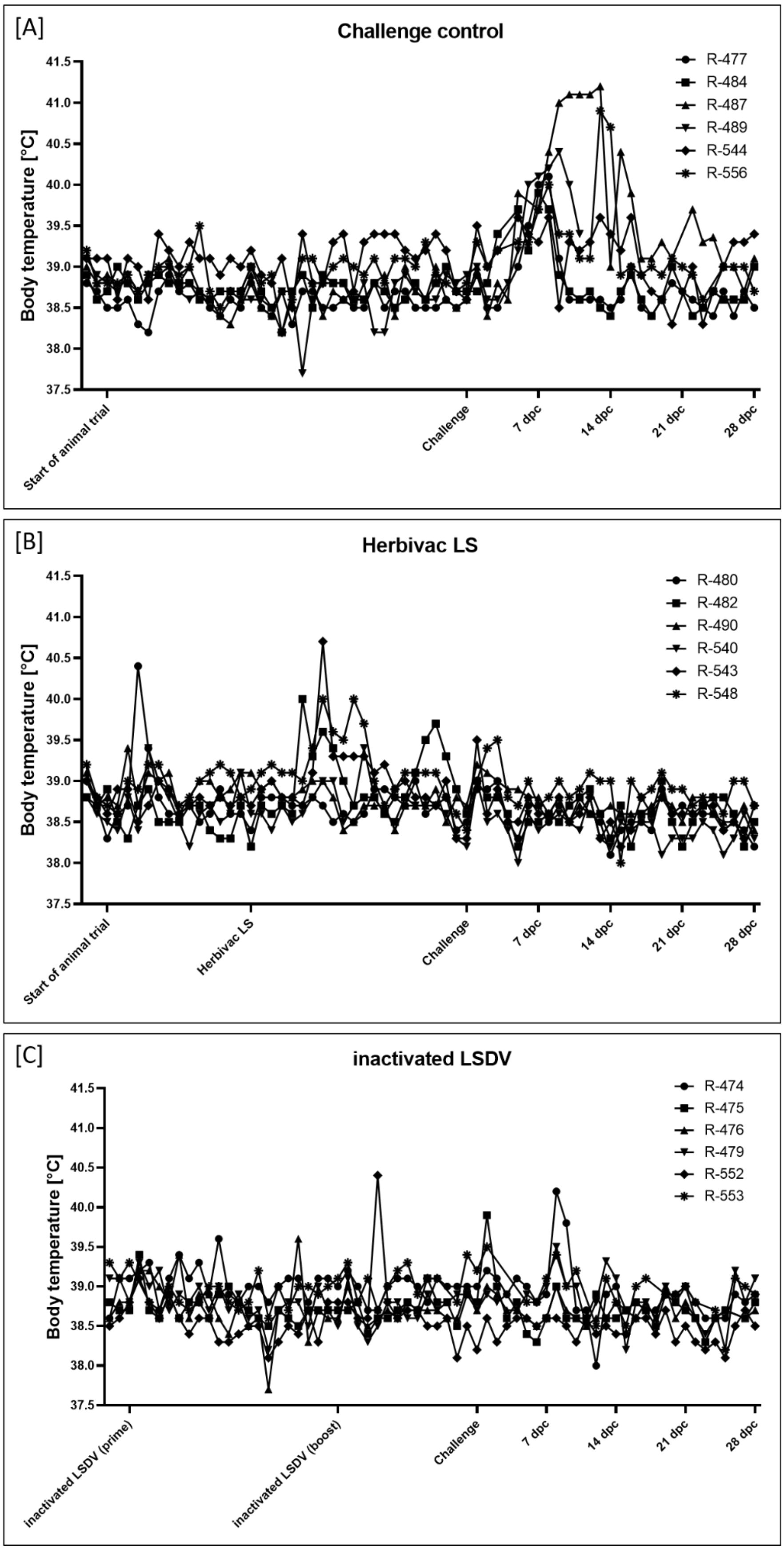

Supplemental Figure S1. Body temperature of the cattle during the proof-of-concept animal trial. Body temperature was measured daily starting a few days before first vaccination with inactivated LSDV until 28 dpc. (**A**) Cattle of Group 1A were left unvaccinated and served as challenge control group. (**B**) Cattle of Group 1B received the commercially available life-attenuated vaccine "Herbivac LS", and (**C**) cattle of Group 1C were vaccinated with an inactivated vaccine prototype on basis of LSDV-"Neethling vaccine" strain.

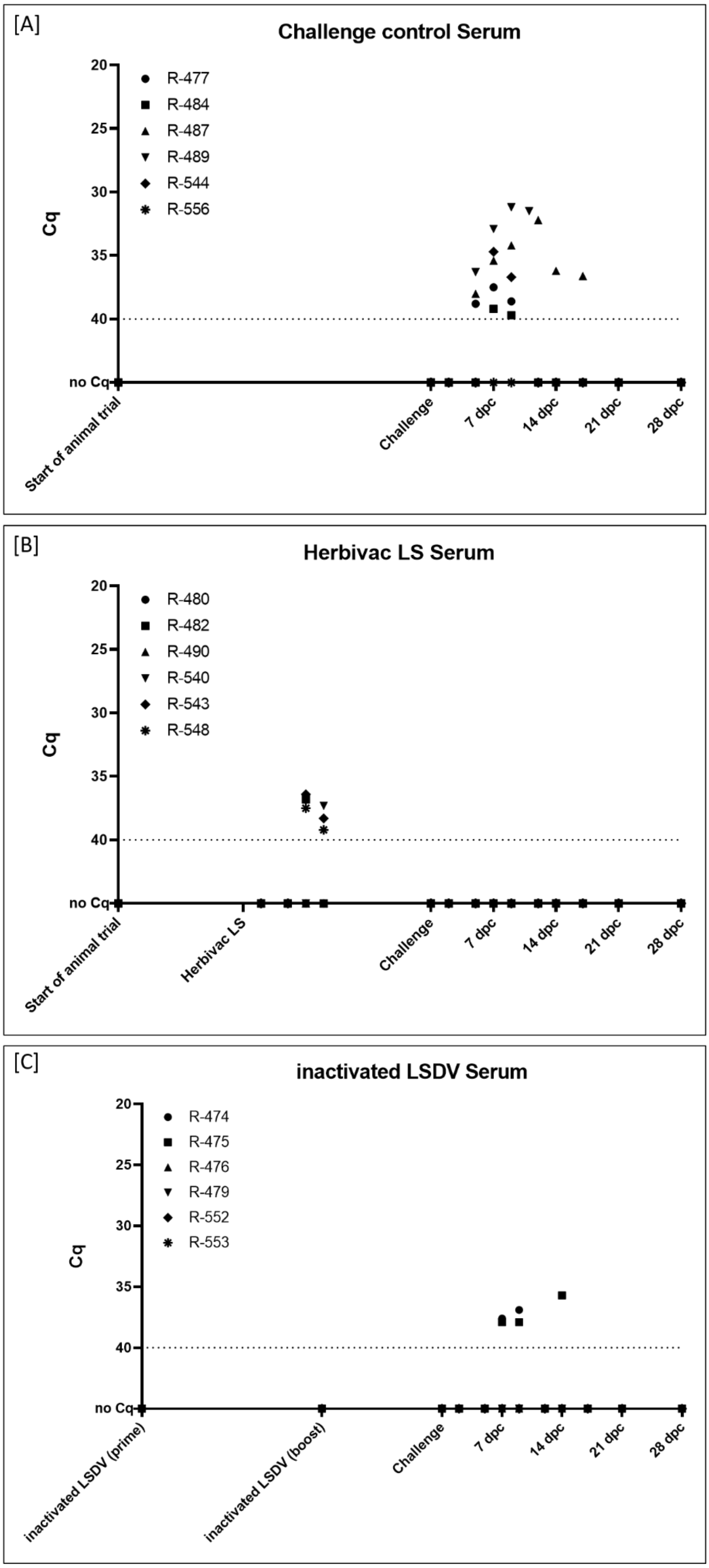

Supplemental Figure S2. Viral genome load in serum samples taken during the proof-of-concept study. (**A**) Cattle of Group 1A were left unvaccinated and served as challenge control group. (**B**) Cattle of Group 1B received the commercially available life-attenuated vaccine "Herbivac LS", and (**C**) cattle of Group 1C were vaccinated with an inactivated vaccine prototype on basis of LSDV-"Neethling vaccine" strain.Samples were taken at defined time points during the animal trial and analyzed regarding their viral genome load. Cut-off was defined at Cq 40.0.

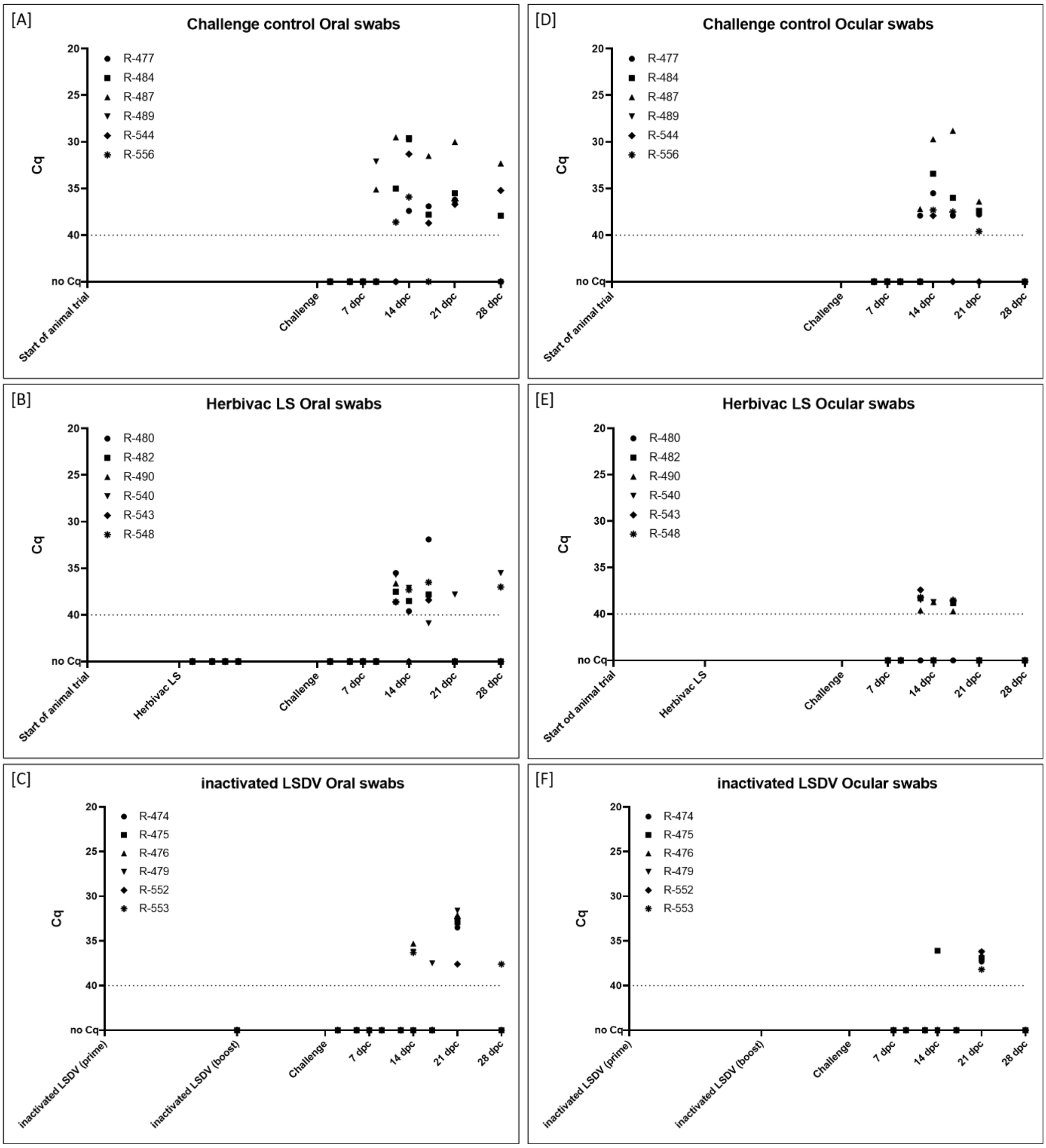

Supplemental Figure S3. Viral genome load in (**A**-**C**) oral and (**D**-**F**) ocular swab samples taken during the proof-of-concept study. (**A**+**D**) Cattle of Group 1A were left unvaccinated and served as challenge control group. (**B**+**E**) Cattle of Group 1B received the commercially available life-attenuated vaccine "Herbivac LS", and (**C**+**F**) cattle of Group 1C were vaccinated with an inactivated vaccine prototype on basis of LSDV-"Neethling vaccine" strain. Samples were taken at defined time points during the animal trial and analyzed regarding their viral genome load. Cut-off was defined at Cq 40.0.

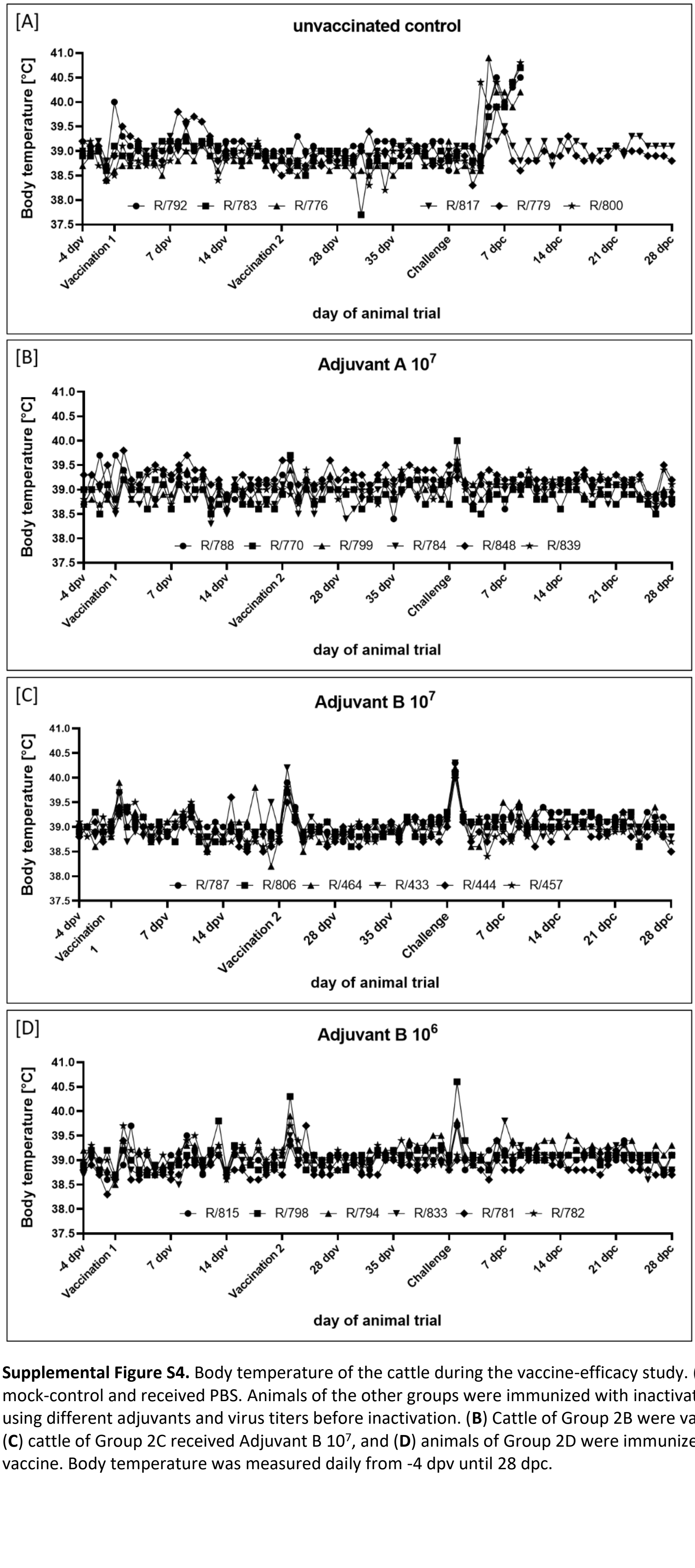

Supplemental Figure S4. Body temperature of the cattle during the vaccine-efficacy study. (**A**) Cattle of Group 2A serve as mock-control and received PBS. Animals of the other groups were immunized with inactivated LSDV-"Serbia" field strain using different adjuvants and virus titers before inactivation. (**B**) Cattle of Group 2B were vaccinated with Adjuvant A 10^7, (**C**) cattle of Group 2C received Adjuvant B 10^7, and (**D**) animals of Group 2D were immunized with Adjuvant B 10^6 prototype vaccine. Body temperature was measured daily from -4 dpv until 28 dpc.

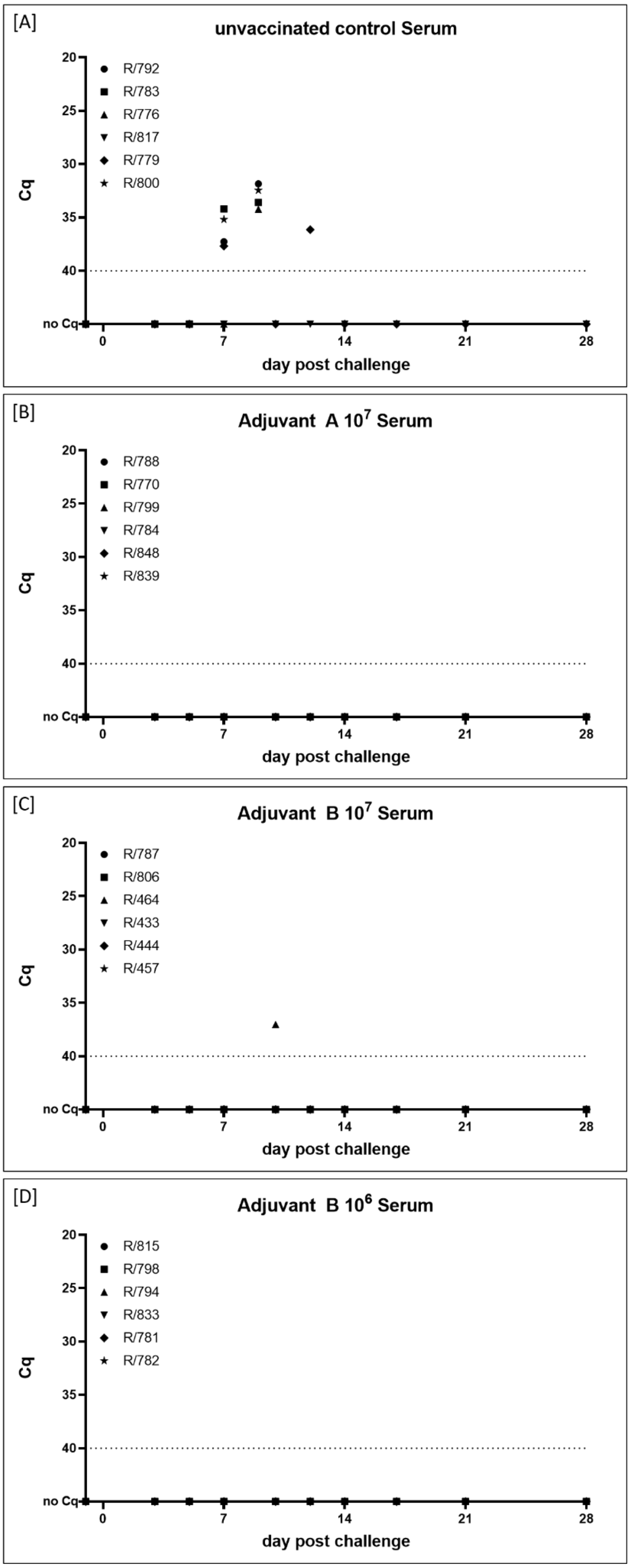

Supplemental Figure S5. Viral genome load in serum samples taken during the vaccine-efficacy animal trial. (**A**) Cattle of Group 2A serve as mock-control and received PBS. Animals of the other groups were immunized with inactivated LSDV-“Serbia” field strain using different adjuvants and virus titers before inactivation. (**B**) Cattle of Group 2B were vaccinated with Adjuvant A 10^7, (**C**) cattle of Group 2C received Adjuvant B 10^7, and (**D**) animals of Group 2D were immunized with Adjuvant B 10^6 prototype vaccine. The samples were taken at certain time points during the animal trial and analyzed regarding the viral genome load. Cut-off was set at Cq 40.0.
